# Generation of a protective murine monoclonal antibody against the stem of influenza hemagglutinins from group 1 viruses and identification of resistance mutations against it

**DOI:** 10.1371/journal.pone.0222436

**Published:** 2019-09-12

**Authors:** Wei Wang, Russell Vassell, Hyo Sook Song, Qiong Chen, Paul W. Keller, Swati Verma, Esmeralda Alvarado-Facundo, Hongquan Wan, Falko Schmeisser, Clement A. Meseda, Jerry P. Weir, Carol D. Weiss

**Affiliations:** Division of Viral Products, Center for Biologics Evaluation and Research, US Food and Drug Administration, Silver Spring, Maryland, United States of America; Shanghai Medical College, Fudan University, CHINA

## Abstract

Vaccines that elicit broadly cross-neutralizing antibodies, including antibodies that target the conserved stem of hemagglutinin (HA), are being developed as a strategy for next-generation influenza vaccines that protect against influenza across multiple years. However, efficient induction of cross-neutralizing antibodies remains a challenge, and potential escape mutations have not been well characterized. Here we elicited cross-neutralizing antibodies by immunizing animals with the hemagglutinins from H5 and H9 subtype influenza A viruses that are sensitive to neutralization by stem antibodies. We further isolated and characterized an HA stem monoclonal antibody 4C2 that broadly neutralizes group 1 influenza viruses and identified HA mutations that reduced sensitivity to stem antibodies. Our results offer insights for next-generation influenza vaccine strategies for inducing cross-neutralizing antibodies.

## Introduction

Influenza continues to be a global infectious disease threat, causing seasonal epidemics and occasional pandemics due to the emergence of an influenza A virus containing a hemagglutinin (HA) subtype that has not recently circulated in humans. Humoral immune responses against the HA protein, the principal antigen in inactivated influenza vaccines, correlate with protection against influenza. Therefore, vaccination provides an important public health strategy.

HA is comprised of the HA1 surface subunit, forming the globular head domain that mediates binding to cell surface sialic acid receptors, and the HA2 transmembrane subunit, forming the major part of the stem region that mediates membrane fusion between viral and endosomal membranes during endocytosis. HA1 and HA2 are disulfide linked and make additional non-covalent interactions between the N- and C-termini of HA1 and the ectodomain of HA2 in HA stem region. Most neutralizing antibodies elicited by influenza virus infection or vaccination target the receptor binding site and surrounding residues on the HA1 head domain [1, 2]. Viruses readily mutate these residues to escape antibody neutralization, leading to high sequence variability in the HA1 head domain. Thus, neutralizing antibodies targeting head epitopes are usually strain specific [3, 4]. Due to the frequent emergence of influenza variants with mutations in HA that change antigenicity, influenza vaccines are reformulated annually to match the dominant circulating strains.

Recently, broadly neutralizing antibodies targeting the HA stem have been discovered [5–14]. The HA stem region is highly conserved within influenza groups. Thus, the stem region is an attractive target for developing next-generation influenza vaccines that elicit broadly neutralizing stem antibodies. However, neutralizing stem antibodies in humans generally do not reach high titers after infection or vaccination [15], and ways to efficiently induce neutralizing stem antibodies remain a major challenge.

We and others previously showed that different virus strains, even those within the same subtype with identical stem epitopes, may have different susceptibilities to neutralization by stem antibodies and cross-neutralizing sera [8, 16, 17]. This suggests that HA epitopes for cross-neutralizing antibodies from sensitive viruses are better exposed for binding to cross-neutralizing antibodies. In this study, we investigated cross-neutralizing antibody responses induced by HAs from virus strains that are sensitive to HA stem antibody neutralization. We isolated and characterized one neutralizing stem monoclonal antibody, 4C2, and identified HA mutations that allowed viral escape. Our results have implications for next-generation influenza vaccination strategies intended to induce cross-neutralizing antibodies.

## Materials and methods

### Ethics

Animals were housed at the Division of Veterinary Services in the FDA White Oak animal facility in Silver Spring, MD. Experiments were performed under protocols numbers 2009–28 and 2014–04, approved by the US Food and Drug Administration Institutional Animal Care and Use Committee.

### Cells and viruses

Human 293T cells and Madin-Darby Canine Kidney (MDCK) cells were maintained in Dulbecco’s Modified Eagle’s Medium (DMEM) with high glucose, L-glutamine, Eagle′s minimum essential medium (MEM) non-essential amino acids, penicillin/streptomycin, 4-(2-hydroxyethyl)-1-piperazineethanesulfonic acid (HEPES) buffer, and 10% fetal calf serum. Influenza viruses (A/Puerto Rico/8/1934) were generated by reverse genetics as described previously [18, 19]. Briefly, 1 μg each of pHW2000 plasmids containing each of the eight genes of A/Puerto Rico/8/1934 virus [20] (kindly provided by Maryna Eichelberger, US Food and Drug Administration (FDA), Silver Spring, MD) were transfected into a mixture of 293T and MDCK cells. The transfection cocktail was replaced with Opti-MEM I medium (Invitrogen, Grand Island, NY) after 6 h of incubation at 37°C. Opti-MEM I medium supplemented with trypsin (1 μg/ml) (Sigma-Aldrich, St. Louis, MO) was added 24 h later. The culture supernatant was collected at 48 to 72 h post-transfection. The viruses were then propagated and titered by plaque assay on MDCK cells for isolation of escape mutants.

To generate an escape mutant, H1N1 A/Puerto Rico/8/1934 viruses were incubated with the monoclonal antibody (MAb) over a range of concentrations from 60–1 μg/ml in a total volume of 0.6 ml for 1 h at room temperature, followed by infection of ~ 2.3 x 10^6^ MDCK cells with the virus-antibody mixtures. After a 1.5 h adsorption, virus inoculum was removed, and the cells were overlaid with serum-free media containing 2 μg/ml L-1-tosylamide-2-phenylethyl chloromethyl ketone (TPCK)-treated trypsin and MAb at the desired concentration. Virus recovered at the highest concentration of MAb underwent five additional rounds of selection. Potential escape mutant viruses were tested for neutralization resistance. Consensus nucleotide sequences of viral HAs from resistant viruses were determined by direct DNA-sequencing of reverse transcription-polymerase chain reaction (RT-PCR) products and compared with those of the parental, wild-type virus.

### HAs and MAbs

Recombinant HAs of A/Singapore/1/1957 (H2), and A/chicken/Hong Kong/G9/1997 (H9) were obtained from the Biodefense and Emerging Infections Research Resources Repository (BEI Resources). HA of A/Vietnam/1203/2004 (H5) was from an inactivated H5N1 influenza virus vaccine (Sanofi Pasteur). Anti-A/Vietnam/1203/2004 HA head neutralizing MAb VN2.4 and anti-A/Vietnam/1203/2004 HA head non-neutralizing MAb VN2.5 (both IgG1) were provided by Jerry P. Weir (unpublished data). Anti-A/Puerto Rico/8/1934 HA head neutralizing MAb H17-L2 [21–23] was kindly provided by Scott Hensley (Wistar Institute, Philadelphia, PA). The HIV-1 gp41 antibody (Chessie 8) [24] was obtained from the National Institutes of Health AIDS Research and Reference Reagent Program. Broadly neutralizing stem MAbs CR6261 and FI6 [5, 7] were obtained from the Vaccine Research Center (NIH, Bethesda, MD) and C179 [11] was kindly provided by Yoshinobu Okuno (Kyoto, Japan).

### HA-pseudovirus production, HA-pseudovirus neutralization, and plaque reduction neutralization

Full-length HA ORFs from H1, H2, H3, H5, H7 and H9 viruses (Table 1), and NA ORF from A/California/04/2009 (H1N1) (GenBank FJ966084) were either amplified from viruses by RT-PCR or chemically synthesized (GenScript, Piscataway, NJ), placed into the pCMV/R expression plasmid obtained from Dr. Gary J. Nabel (National Institutes of Health (NIH), Bethesda, MD), as described previously [25], and then used for HA-pseudovirus production. HA-pseudoviruses carrying a luciferase (Luc) reporter gene were produced in 293T cells as described previously [25, 26]. Briefly, 5 μg of pCMVΔR8.2, 5.5 μg of pHR’CMVLuc, 2.5 μg of HAT [27, 28], 0.5 μg of HA (codon optimized) or 4 μg of HA (non-codon optimized) and 4 μg of A/California/04/2009 NA expression plasmids were included in the transfection. In addition, 2.5 μg of an A/Puerto Rico/8/1934 M2 expression plasmid [29] were added in transfection to produce A/Mexico/4108/2009 HA-pseudoviruses [30]. HA-pseudoviruses were collected 48 h post-transfection, filtered through a 0.45 μm low protein binding filter and used immediately or stored at -80°C. HA-pseudovirus titers were measured by infecting 293T cells with HA-pseudoviruses for 48 h prior to measuring luciferase activity in infected cells (luciferase assay reagent, Promega, Madison, WI) as described previously [25]. HA-pseudovirus titers were expressed as relative luminescence unit per milliliter of HA-pseudovirus supernatants (RLU/ml).

**Table 1:** HA gene list.

Virus	HA subtype (Genbank or GISAID ID)
A/Mexico/4108/2009	H1 (pdm09) (GQ223112)
A/Puerto Rico/8/1934	H1 (CY009444)
A/Beijing/262/95	H1 (AY289928)
A/New Caledonia/20/1999	H1 (AY289929)
A/Brisbane/59/2007	H1 (CY058487)
A/Japan/305/1957	H2 (DQ508841)
A/swine/Missouri/4296424/2006	H2 (EU258943)
A/Philippines/2/82	H3 (CY121245)
A/Vietnam/1203/2004	H5 (EF541403)
A/Indonesia/5/05	H5 (EF541394)
A/duck/Guangxi/1378/2004	H5 (DQ320884)
A/duck/Hubei/wg/2002	H5 (DQ997094)
A/chicken/Vietnam/NCVD-016/2008	H5 (FJ842476)
A/Shanghai/02/2013	H7 (EPI439502)
A/Hong Kong/1073/99	H9 (AJ404626)
A/chicken/Hong Kong/G9/97	H9 (AF156373)

Abbreviation: GISIAD, Global Initiative on Sharing All Influenza Data

HA-pseudovirus neutralization was performed as described previously [25–27, 30, 31]. Briefly, HA-pseudoviruses that deliver approximately 1x10^6^ RLU of luciferase activity were incubated with antibodies or sera for 1 h at 37°C. Pseudovirus and antibody mixtures (100 μl) were then inoculated onto 96-well plates that were seeded with 2.5 x 10^4^ 293T cells/well one day prior to infection. HA-pseudovirus infectivity was evaluated 48 h later in the luciferase assay. The antibody dilution causing a 95% reduction of RLU compared to control (IC95-neutralizing antibody titer) was used as the neutralization endpoint titer [27]. IC95 was calculated using a nonlinear regression curve fit (Graphpad Prism software). The mean of IC95 from at least two independent experiments was reported as final IC95 titer.

As described previously [32], plaque reduction neutralization (PRN) assay was performed in MDCK cells with a 50:50 mixture of 2X EMEM plaque media (BioWhittaker, Walkersville, MD) and 2.4% Avicel RC-581 (FMC BioPolymer, Philadelphia, PA) in the presence of TPCK-treated trypsin (Sigma-Aldrich Corp. St. Louis, MO) at a final concentration of 2 μg/ml. The antibody causing a 50% reduction of plaque compared to control (IC50-neutralizing antibody titer) was used as the neutralization endpoint titer. IC50 was calculated using a nonlinear regression curve fit (Graphpad Prism software). The mean of IC50 from at least two independent experiments was reported as final PRN titer.

### Animal immunizations and generation of mouse hybridomas secreting HA-specific MAbs

Two New Zealand white rabbits were primed subcutaneously with 75 μg of A/chicken/Hong Kong/G9/1997 (H9) HA (BEI Resources) mixed with Freund’s complete adjuvant (Becton Dickinson, Sparks, MD), followed by two boosts of 75 μg of A/chicken/Hong Kong/G9/1997 (H9) HA mixed with Freund’s incomplete adjuvant (Becton Dickinson, Sparks, MD) and two boosts of 45 μg of A/Vietnam/1203/2004 (H5) HA (Sanofi Pasteur) mixed with Freund’s incomplete adjuvant, at four-week intervals. Serum samples collected pre-immunization and 4 weeks after each immunization were heated to 56°C for 30 min to inactivate complement for the neutralization assay.

Mouse hybridomas secreting HA-specific MAbs were prepared by Precision Antibody (Columbia, MD). Briefly, BALB/c mice were immunized intraperitoneally with 18 μg of A/chicken/Hong Kong/G9/1997 (H9) HA mixed with Freund’s complete adjuvant, followed by one boost of 18 μg of A/chicken/Hong Kong/G9/1997 (H9) HA mixed with Freund’s incomplete adjuvant, two boosts of 9 μg of A/Vietnam/1203/2004 (H5) HA mixed with Freund’s incomplete adjuvant, and one boost of 10 μg of A/chicken/Hong Kong/G9/1997 (H9) HA and 10 μg of A/Singapore/1/1957 (H2) HA (BEI Resources) mixed with Freund’s incomplete adjuvant, at two-week intervals. Serum samples collected pre-immunization and two weeks after each immunization were heated to 56°C for 30 min for neutralization assays. Splenocytes from immunized mice were fused with mouse myeloma cells Sp2/0, and the hybridomas were screened for H5, H2 and H9 HA-specific MAbs secretion by enzyme-linked immunosorbent assay (ELISA) using HA proteins. Positive hybridomas were subcloned to verify clonality. The heavy and light chain genes of MAbs were sequenced and cloned into expression plasmids by GenScript (Piscataway, NJ). Both IgG1 and IgG2a isotypes of MAb were expressed and purified by GenScript.

### MAb binding to HA by ELISA

To screen MAbs for binding to influenza virus HA, HA antigen (10 μg/ml) was coated onto ELISA plates. The coated plates were blocked and incubated with MAbs. The plates were then washed with PBS (pH 7.2) and incubated with peroxidase-conjugated secondary antibody (KPL, Gaithersburg, MD). Unbound secondary antibody was washed off with PBS (pH 7.2), signal was developed using 3,3′,5,5′-Tetramethylbenzidine (TMB) substrate, and the reaction was stopped with 1M H_2_SO_4_ before recording OD_450_. The endpoint titer was defined as the highest dilution that gave an absorbance value greater than 0.050.

Competition ELISAs were used to determine whether the new cross-neutralizing MAbs overlapped other known neutralizing antibodies. H5 HA antigen (2 μg/ml) was coated onto ELISA plates. After blocking, the coated plates were incubated with mixtures of biotinylated MAb 4C2 (final concentration 1μg/ml) and other antibodies at various concentrations. The plates were then washed with Tris-buffered saline (TBS)-0.05% Tween-20 and incubated with horseradish peroxidase-conjugated streptavidin (KPL, Gaithersburg, MD). Signal was developed, and OD_450_ was recorded.

### Mouse challenge study

Six week old BALB/c mice (Jackson Laboratory, Bar Harbor, ME) were immunized with 9 μg of A/chicken/Hong Kong/G9/1997 (H9) HA mixed with TiterMax adjuvant (Sigma-Aldrich, St. Louis, MO), followed by 1 boost of 9 μg of A/Vietnam/1203/2004 (H5) HA mixed with TiterMax adjuvant, and 1 boost of 9 μg of A/Singapore/1/1957 (H2) HA mixed with TiterMax adjuvant by subcutaneous injection, at 2-week intervals. Two weeks after the final boost, mice were anesthetized and challenged intranasally with 5 50% lethal dose (LD_50_) of A/Puerto Rico/8/1934 virus. Body weights were monitored daily and mice that reached a study endpoint of 25% loss in body weight were humanely euthanized using CO_2_. In passive MAb transfer experiments, mice were administered intraperitoneally with MAbs 6 h before challenge with 5 LD_50_ of A/Puerto Rico/8/1934 virus.

### Immunoprecipitation

Whole viral particles and purified HA proteins have been used to demonstrate acidic pH-induced HA conformation changes [29, 33–38]. The H1 HA-pseudoviruses, made in serum-free medium in the absence of human airway trypsin-like protease (HAT), were used for MAb binding. In brief, HA-pseudovirus supernatant samples were mixed with 1 M citrate buffers with different pH and 10% n-dodecyl β-D-maltoside (DDM) to final concentrations of 0.1 M citrate and 1% DDM, followed by incubation at 37°C for 1 h. The samples were neutralized with 1 M Tris (pH 8) and then incubated with stem MAb at 37°C for another 1 h, followed by immunoprecipitation with protein G Dynabeads (Invitrogen) overnight at 4°C as previously reported [39]. After immunoprecipitation, the protein G beads were washed four times with 1% NP40-PBS. The immunoprecipitation samples were resolved on sodium dodecyl sulfate–polyacrylamide gel electrophoresis (SDS-PAGE) and detected by Western blot using rabbit antiserum against H1 HA2 subunit [38].

### Computational analysis

The interactions between escape mutation residues and their neighbor residues were modeled with the UCSF Chimera program (http://www.cgl.ucsf.edu/chimera/) using the Protein Data Bank (PDB) entries 1RU7, 3ZTN, 3GBN, 4HLZ, 3ZTJ, 3GBM.

## Results

### Heterosubtypic HA immunizations induced protective, heterosubtypic cross-neutralizing antibody

We selected HA immunogens from A/Singapore/1/1957 (H2), A/Vietnam/1203/2004 (H5), and A/chicken/Hong Kong/G9/1997 (H9) that we previously found to be sensitive to cross-neutralizing antibodies [16, 17]. Higher sensitivity to stem antibodies raised the possibility that stem epitopes on these HAs were better exposed. We analyzed neutralization with HA-pseudoviruses. The increased sensitivity and larger dynamic range of the HA-pseudovirus neutralization assay compared to the plaque reduction neutralization assay, particularly for stem-directed HA antibodies, allows greater discrimination of the neutralization phenotype among HAs [40].

**Fig 1:**
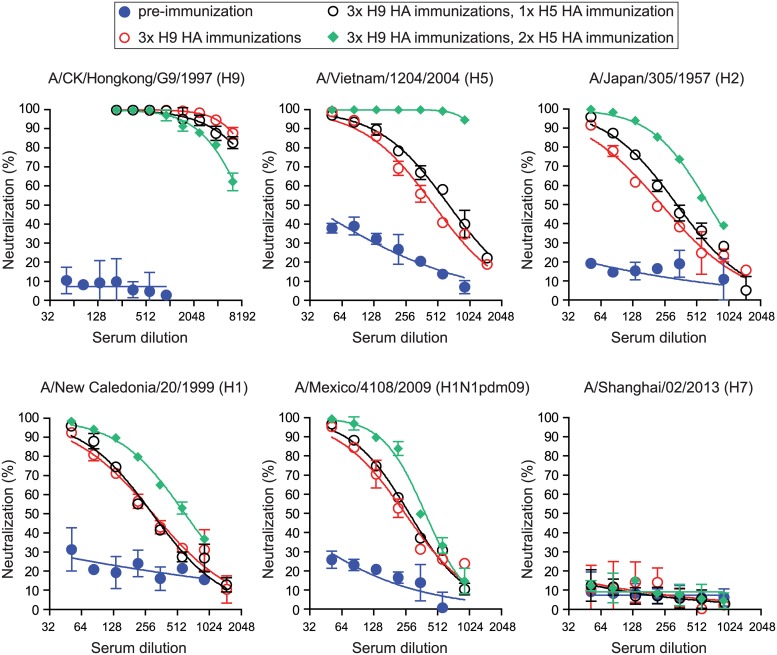
Heterosubtypic HA immunizations elicit cross-neutralizing antibodies. Neutralization ability against A/chicken/Hong Kong/G9/1997 (H9), A/Vietnam/1203/2004 (H5), A/Japan/305/1957 (H2), A/New Caledonia/20/1999 (H1), A/Mexico/4108/2009 (H1N1pdm09) and A/Netherland/219/2003 (H7) HA-pseudoviruses in one rabbit sera collected pre- and post- immunizations as indicated. Data are shown as means and standard deviations. Blue circle: pre-immunization; Red open circle: post 3 immunizations of H9 HA; Black open circle: post 3 immunizations of H9 HA and one immunization of H5 HA; Green diamond: post 3 immunizations of H9 HA and two immunizations of H5 HA. Similar results were obtained in other rabbit immunizations.

We first immunized two rabbits with A/chicken/Hong Kong/G9/1997 (H9) HA three times, followed by two immunizations with A/Vietnam/1203/2004 (H5N1) vaccine. While the pre-immunization sera did not have neutralization titers to all tested H1, H2, H5, H7 and H9 HA-pseudoviruses (Fig 1), rabbits immunized three times with H9 HA immunizations induced high neutralizing antibody titers to A/chicken/Hong Kong/G9/1997 HA (H9) and modest, cross-neutralizing antibody titers to A/Vietnam/1203/2004 (H5) HA, A/Japan/305/1957 (H2) HA, A/New Caledonia/20/1999 (H1) and A/Mexico/4108/2009 (H1N1pdm09) HA, but no cross-neutralizing antibodies to A/Shanghai/02/2013 (H7) HA (Fig 1). Two additional H5 HA immunizations further boosted neutralization titers to A/Vietnam/1203/2004 (H5) HA and cross-neutralization titers to A/Japan/305/1957 (H2) HA, A/New Caledonia/20/1999 (H1) and A/Mexico/4108/2009 (H1N1pdm09) HA, but not to A/Shanghai/02/2013 (H7) HA (Fig 1). These data show that H9 and H5 HAs from sensitive virus strains can elicit cross-neutralizing antibody against heterosubtypic HA in naïve animals.

**Fig 2:**
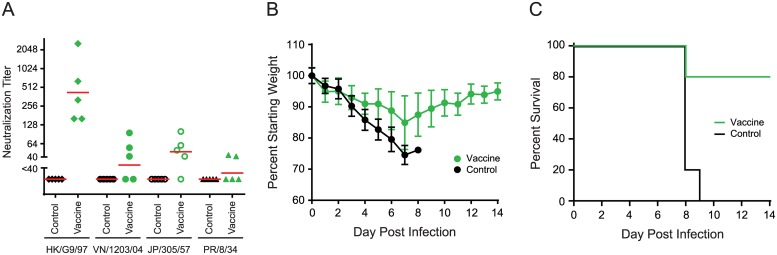
Heterosubtypic HA immunizations provide cross-protection against influenza virus challenge. Mice (5 per group) were immunized once with A/chicken/Hong Kong/G9/1997 (HK/G9/97) (H9) HA, followed by one boost with A/Vietnam/1203/2004 (VN/1203/04) (H5) HA and one boost with A/Singapore/1/1957 (H2) HA, then challenged with 5 LD_50_ A/Puerto Rico/8/1934 (PR/8/34). Mice were observed for 2 weeks for body weight loss and/or death after challenge. (A) Neutralization titers of mice sera post-immunization. (B) Average percentage change in body weight post-challenge. Bars: standard deviations. (C) Mice survival curves post-challenge. Control: mock immunization (without influenza HA); Vaccine: immunization with influenza HA.

To further evaluate our immunization strategy and the protective ability of cross-neutralizing antibodies against heterosubtypic influenza viruses challenge, we immunized mice with recombinant HAs for A/chicken/Hong Kong/G9/1997 (H9) and A/Singapore/1/1957 (H2) and split, inactivated A/Vietnam/1203/2004 (H5) vaccine. We then challenged with a lethal dose of A/Puerto Rico/8/1934 (H1N1). Mice vaccinated with H9, H5, and H2 antigens generated not only strain-specific neutralizing antibodies against A/chicken/Hong Kong/G9/1997 (H9) HA, A/Vietnam/1203/2004 (H5) HA, and A/Japan/305/1957 (H2) HA, but also low-level cross-neutralizing antibodies against A/Puerto Rico/8/1934 (H1) HA in two of five mice (Fig 2A). Importantly, H9, H5 and H2 HA vaccinations significantly protected 80% of the mice against a heterosubtypic A/Puerto Rico/8/1934 (H1N1) lethal-dose challenge, as measured by weight loss and survival, while unvaccinated mice were not protected (Fig 2B and 2C). HA antibodies likely account for most of the protection, but we cannot rule out that residual amounts of non-HA viral proteins that may be present in the H5 vaccine may have contributed to protection.

### Cross-neutralizing stem MAb generation and characterization

We next tried to isolate MAbs that may have contributed to the cross-protection. We repeated our immunization strategy in mice and selected hybridomas that secreted MAbs against H9, H5, and H2 HAs. Among these hybridomas, clone 4C2 was found to produce a MAb that neutralized H9, H5, H2 and H1 HA-pseudoviruses, but not H3 and H7 HA-pseudoviruses (Table 2). However, MAb 4C2 had undetectable hemagglutination inhibition (HI) activity against these viruses. Since the HA-pseudovirus assay is a single-cycle infection assay, only neutralization at the entry stage was measured.

**Table 2:** Summary of MAb 4C2 neutralization titers.

Virus	Neutralization titer (μg/ml)	
	IgG1	IgG2a
A/Mexico/4108/2009 (H1)	1.90	1.84
A/Puerto Rico/8/1934 (H1)	0.60	0.68
A/Beijing/262/95 (H1)	0.38	0.40
A/New Caledonia/20/1999 (H1)	0.92	0.88
A/Brisbane/59/2007 (H1)	6.00	ND
A/Japan/305/1957 (H2)	0.41	0.44
A/swine/Missouri/4296424/2006 (H2)	12.61	ND
A/Philippines/2/82 (H3)	> 50.00	> 50.00
A/Vietnam/1203/2004 (H5)	0.24	0.31
A/Indonesia/5/05 (H5)	1.20	1.11
A/duck/Guangxi/1378/2004 (H5)	7.35	ND
A/duck/Hubei/wg/2002 (H5)	7.37	7.01
A/chicken/Vietnam/NCVD-016/2008 (H5)	3.87	ND
A/Shanghai/02/2013 (H7)	> 50.00	> 50.00
A/Hong Kong/1073/99 (H9)	0.77	0.72
A/chicken/Hong Kong/G9/97 (H9)	0.74	0.76

Abbreviation: ND, Not Done

**Fig 3:**
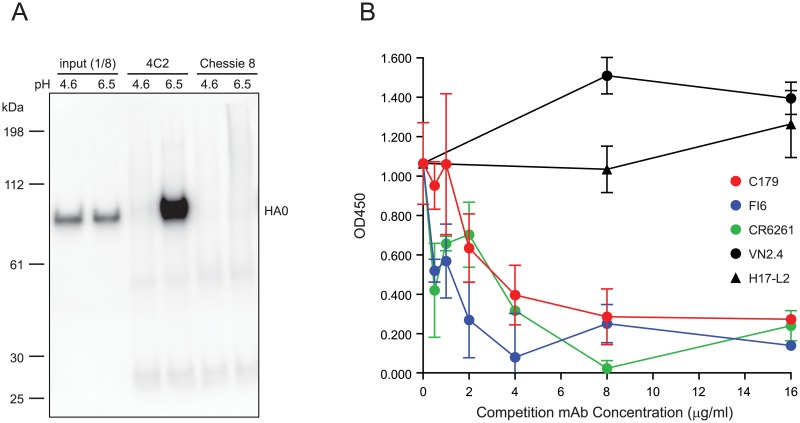
MAb 4C2 binds to HA with native conformation and recognizes the epitopes targeted by stem antibodies. (A) MAb 4C2 binds to HA with native conformation. A/New Caledonia/20/1999 (H1) HA-pseudoviruses (HAT was not included in HA-pseudoviruses production, and thus HA presented as HA0) were treated under different pH conditions, then immunoprecipitated with MAb 4C2 or Chessie 8, and analyzed by Western blotting with antiserum against H1 HA2. Input: 1/8 of total sample used for immunoprecipitation; MAb Chessie 8: Negative immunoprecipitation control. (B) Stem antibodies compete against MAb 4C2 binding to HA. MAb 4C2 binding to H5 HA was competed with HA head antibodies (VN2.4 and H17-L2) and stem antibodies (C179, FI6, and CR6261) in a competition ELISA. Data are shown as means and standard deviations.

To characterize MAb 4C2, we performed immunoprecipitation and competition ELISAs to detect MAb 4C2 binding to HA. Immunoprecipitation studies showed that MAb 4C2 binds to the neutral pH conformation of HA, but not to the low pH HA conformation (Fig 3A). When MAb 4C2 (1 μg/ml) was competed with various concentrations of HA head antibodies (MAbs VN2.4 and H17-L2) and stem antibodies MAbs C179, FI6, and CR6261, competition ELISA results showed that stem antibodies, but not HA head antibodies, competed against MAb 4C2 binding to H5 HA (Fig 3B), suggesting that the epitopes recognized by MAb 4C2 and stem MAbs C179, CR6261, and FI6 may share some of the same residues. Taken together, these data indicate that MAb 4C2 interacts with the HA stem region, similar to MAbs C179, CR6261, and FI6.

Sequencing of MAb 4C2 showed that it was an IgG1 isotype (S1 Fig). Since the *in vivo* protection of neutralizing stem antibody has been largely associated with Fc-mediated activation of humoral and cellular innate immune functions, and thus influenced by IgG isotypes [41, 42], we also converted MAb 4C2-IgG1 to MAb 4C2-IgG2a for protection studies (below). However, as noted, both IgG2a and IgG1 isotypes of MAb 4C2 have same neutralization titers *in vitro* (Table 2).

### Protection afforded by passive transfer of MAb 4C2

**Fig 4:**
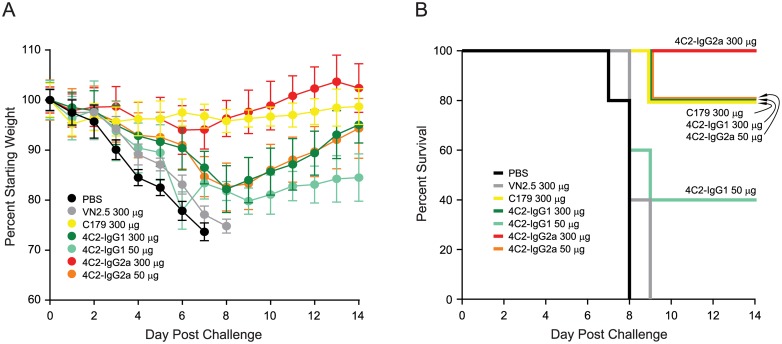
MAb 4C2 protects mice from influenza challenge. Mice (5 per group) were inoculated intraperitoneally with PBS or the indicated MAb doses 6 h before being challenged with 5 LD_50_ of A/Puerto Rico/8/1934 virus. Mice were observed 2 weeks for body weight loss and/or death. (A) Average percentage change in body weight post-challenge. Bars: standard deviations. (B) Survival curves post-challenge. Similar results were obtained in two additional independent challenge experiments.

To assess the protective capacity of MAb 4C2, mice were injected intraperitoneally with high and low doses of this antibody prior to challenge with a lethal dose of A/Puerto Rico/8/1934 (H1N1). As controls, PBS or an H5N1 HA MAb VN2.5 failed to protect mice from morbidity or mortality (Fig 4). The greatest weight loss was observed in the PBS and MAb VN2.5 groups, with weight loss persisting for 7–8 days until mice died or were sacrificed when they reached the study endpoint (Fig 4A). In contrast, like the control stem MAb C179, MAb 4C2 IgG1 isotype provided substantial protection against challenge (Fig 4B). Both 300 μg (approximately 14 mg MAb per kg mouse) of MAb C179 (IgG1) and MAb 4C2-IgG1 protected mice from death with only 20% mortality. However, weight loss in the MAb 4C2-IgG1 group was greater than that in MAb C179 group. At a dose of 50 μg (approximately 2.3 mg MAb per kg mouse), MAb 4C2-IgG1 protected mice less efficiently (40% survival), with mice having more weight loss and slower weight recovery. Since stem antibodies of IgG2a isotype have been reported to provide better protection than the IgG1 isotype [41], we also compared IgG1 to IgG2a isotypes of 4C2 for their ability to provide passive protection. As expected, MAb 4C2 IgG2a provided much better protection against viral challenge (Fig 4). Passive transfer of 300 μg (approximately 14 mg MAb per kg mouse) of MAb 4C2-IgG2a protected 100% of the mice from death and resulted in less weight loss and a quicker weight recovery. Interestingly, a 50 μg (approximately 2.3 mg MAb per kg mouse) dose of MAb 4C2-IgG2a provided the same level of protection as did 300 μg of MAb 4C2-IgG1.

### Escape mutant viruses to MAb 4C2

We next generated escape mutant viruses to MAb 4C2 by culturing A/Puerto Rico/8/1934 (H1N1) virus in MDCK cells in the presence of MAb 4C2. After a dozen attempts and extensive passaging, we obtained an escape mutant virus with a lysine to glutamic acid substitution at position 55 (K55E) (H3 HA numbering, without signal peptide) in HA1 and a valine to leucine substitution at position 444 (V444L) (H3 HA numbering or V115L in HA2 numbering) in HA2 that conferred low-level resistance to MAb 4C2. Additional resistance mutations would be needed for higher levels of resistance, and compensatory mutations might be needed to maintain fitness *in vivo*. A second escape mutant isolate carried only the V444L substitution in HA. The control virus cultured without the presence of MAb 4C2 did not show any HA mutations.

To determine whether these mutations conferred resistance to stem antibodies, we engineered viruses and HA-pseudoviruses with an HA gene containing single mutations and both mutations. Both wild type and the engineered escape mutant viruses replicate well in MDCK cells and have similar infectivity, suggesting no *in vitro* fitness cost for the escape mutant viruses. Since escape mutant viruses were selected by culturing virus in the presence of MAb 4C2 in MDCK cells, we further included the plaque reduction neutralization (PRN) assay that uses replicating viruses to evaluate the sensitivity of escape mutants to MAb 4C2. We found that PRN assay requires much higher concentrations of stem antibodies for neutralization compared to the HA-pseudovirus assay, consistent with prior reports [40]. However, both HA-pseudovirus neutralization and PRN assays showed that V444L and K55E together conferred ~7-fold resistance to MAb 4C2 in the HA-pseudovirus assay and ~5-fold resistance in the PRN assay (Table 3). For MAbs C179, CR6261, and FI6 stem antibodies, V444L conferred resistance in both HA-pseudovirus neutralization and PRN assays, while K55E conferred resistance only in HA-pseudovirus neutralization assays but not in PRN assays. The acquisition of K55E and V444L together suggested possible low-level synergy in resistance to MAbs C179 and 4C2 (Table 3). Taken together, V444L appears to play a major role for virus resistance to neutralization by these stem antibodies.

**Table 3:** Stem MAb neutralization against A/Puerto Rico/8/1934 escape mutants.

	HA-pseudovirus neutralization titer (μg/ml)				Plaque reduction titer (μg/ml)		
Mutants	4C2 (IgG1)	C179	CR6261	FI6	4C2 (IgG1)	C179	FI6
WT	0.6	0.31	0.09	0.11	12.8	27.7	3.7
K55E	3.42	2.02	0.43	0.53	35.2	25.7	2.4
V444L	3.01	1.73	0.38	0.49	29.2	68.8	7.1
K55E.V444L	4.53	2.81	0.42	0.54	63.2	91.2	5.9

**Fig 5:**
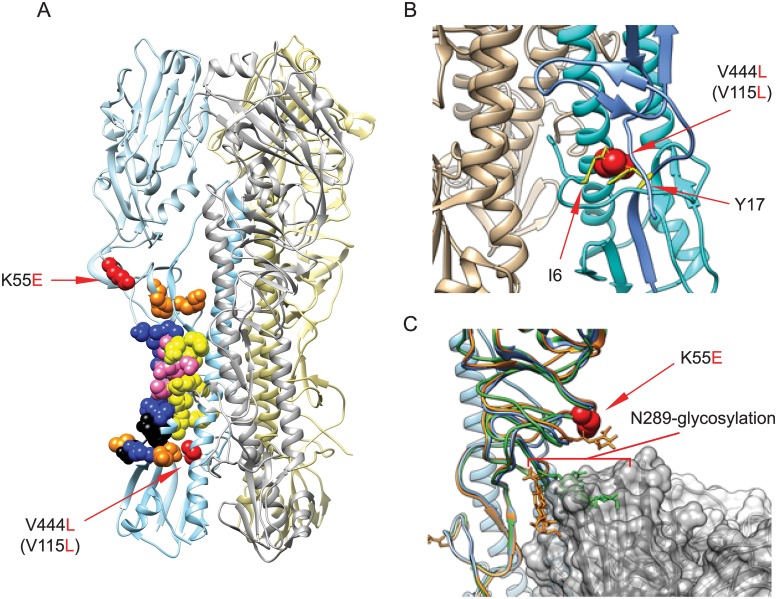
Stem antibody epitopes and MAb 4C2 resistance mutations. (A) The localization of stem antibody epitopes and MAb 4C2 escape mutations in A/Puerto Rico/8/1934 HA trimer is shown. PDB: 1RU7. Yellow: C179 epitopes; Blue: CR6261 epitopes; Orange: FI6 epitopes; Pink: C179 and CR6261 overlap epitopes; Black: CR6261 and FI6 overlap epitopes; Red: MAb 4C2 escape mutations. (B) The changes of mutation V444L (V115L, HA2 numbering) in stem region. V444L (V115L) (red) is in HA2 helix B. The V to L substitution results in van der Waals clashes with residues I6 (HA2) and Y17 (HA1), colored in yellow. (C) The changes of mutation K55E in stem region. K55E (red) is located within a loop region containing HA1 residues 42–58 and 263–314. This region also includes N-linked glycosylation site N289, which has the potential to interfere with stem Ab binding. Shown is MAb FI6 (grey) and aligned HA1 structures from H1 –blue (1RU7), H1+FI6 –orange (3ZTN), and H2 –green (4HLZ). Alignment of HA1 causes N289-glycosylation to clash with the bound antibody in some conformations (green), but not others (orange).

To further understand the nature of these mutations, we modeled the changes using available high-resolution structures. Neither mutation identified in these escape viruses resides directly within the epitopes of well-characterized stem antibodies C179, CR6261, and FI6 (Fig 5A). V444L (or V115L in HA2 numbering) resides in helix B directly behind the helix A stem epitopes of C179, CR6261, and FI6, and adjacent to the cleaved end of the fusion peptide (Fig 5B). Modeling suggests that the introduction of the V444L change results in van der Waals clashes with HA2 fusion peptide residue isoleucine at position 6 (I6) and HA1 residue tyrosine at position 17 (Y17) (Fig 5B), which is directly adjacent to the core stem epitope of C179, CR6261, and FI6 in the 3D structure of H1 HA trimer (PDB: 1RU7). It is likely that this substitution results in structural changes to accommodate these clashes, and these changes may affect the stem epitope conformation and/or availability. Residue K55 is located within a set of loops in HA1 that sits directly above the stem Ab epitope (Fig 5A and 5C), comprised of residues 42 to 58 and 263 to 314. This region includes residues 291–292 contained in the C179 epitope. While modeling of K55 does not reveal direct interactions with the epitope residues of C179, CR6261, and FI6, it may affect exposure and/or conformation of the loops. This region also contains a N-linked glycan site at N289 in the HA stem (Fig 5C), which is occupied in both group 1 and group 2 influenza strains. Molecular modeling on stem antibody-bound HA structures (PDBs: 3ZTN, 3GBN, 4HLZ, 3ZTJ, 3GBM) reveals the potential for steric clash between the glycan at N289 and stem epitope-directed Abs. It is possible that K55E restricts this region’s flexibility to favor conformations in which N289 glycosylation blocks antibody binding.

Altogether, these data and our modeling suggest that MAbs 4C2, C179, CR6261, and FI6 target similar regions in the HA stem, but mutations beyond the epitope may influence fusion peptide function and allosterically affect stem antibody interactions with their epitopes on HA.

## Discussion

The induction of broadly neutralizing antibodies is a goal of many next-generation influenza vaccines that are being developed. Vaccination with or exposure to heterologous antigens as a general strategy for increasing breadth of immune responses to induce anti-HA stem antibodies has been proposed [43–46]. Here we demonstrate in two different animal models that sequential immunizations with heterologous HAs from influenza strains that are sensitive to stem antibody neutralization can induce cross-neutralizing antibodies. Sequential immunizations with H5 and H9 HA in rabbits and H5, H9, and H2 HA in mice elicited broadly neutralizing antibodies that protected mice from a heterosubtypic challenge. We further isolated a monoclonal stem antibody, MAb 4C2, generated with this immunization strategy, and showed that MAb 4C2 also protected mice against a lethal, heterosubtypic, influenza H1N1 challenge.

Similar to the findings of others [41, 42], we found that the IgG isotype affected stem antibody potency. A higher dose of MAb 4C2-IgG1 was needed for protection compared to MAb 4C2-IgG2a. This is likely due to stem antibody interactions with FcγR required for protection against influenza virus *in vivo* [41, 42] since both isoforms of MAb 4C2 had similar *in vitro* potency.

The stem antibody epitopes on HA are very conserved, which may reflect functional constraints on residues in this region and/or limited immune selection against these residues. Generation of escape mutant influenza viruses against MAb 4C2 required many passages, and others have reported that viruses resistant to stem antibodies are not easily generated [13, 14, 47–49]. Although we do not know the exact epitope of MAb 4C2, our competition experiments suggest that MAb 4C2 binds to a similar region in the HA stem as MAbs C179, CR6261, and FI6 [5, 7, 11, 50–54]. Our MAb, which was elicited by a different immunization strategy compared to the other stem MAbs, provides further evidence that this is an immunodominant region in the HA stem. Importantly, we found that MAb 4C2 escape mutations that we identified also contribute to resistance to other stem antibodies.

The escape mutations that emerged in our studies have not been previously identified as contact residues for stem antibodies. Our structural modeling suggests that these residues may alter the fusion peptide, HA stem conformation, and flexibility in a way that affects stem epitope conformation and/or availability and thus influences stem antibody binding. Most reported neutralization escape mutations to stem antibodies are in and around the epitopes of stem antibodies. The major resistance mutations often directly reduce antibody binding affinity with the epitopes [8, 9, 11, 14, 47, 55], while others enhance fusion ability of HA [47, 56]. Recently, Prachanronarong and co-workers reported non-epitope mutations in HA that confer resistance to the stem MAb F10 through the changes of receptor binding, membrane fusion or budding, as well as mutations in NA that allowed virus growth in the presence of the neutralizing antibody [56]. The mutation N460S (N446S, H3 HA numbering, without signal peptide) in their report and our V444L are two residues apart, but both mutations appear to alter the fusion peptide. Although additional studies are needed to elucidate the precise neutralization mechanism of MAb 4C2 and its escape mutants, it appears that virus escape from neutralizing antibodies targeting highly-conserved epitopes in the HA stem may frequently involve mutations in non-epitope residues that are more tolerant of mutations and indirectly affect accessibility or conformation of the neutralizing epitope.

Previously, we reported that conformational stability of HA is an important attribute of susceptibility to broadly neutralizing stem antibodies [17]. HA stability and conformational dynamics likely affect the ability of stem antibodies to access epitopes [48]. Antigen stability has also been reported to control antigen presentation. Thai and coworkers demonstrated that antigen stability affects antigen proteolysis and processing in antigen-presenting cells, T cell stimulation, kinetics of expression of T-cell determinants, and that T-cell stimulation and kinetics of expression of T-cell determinants of antigens correlated inversely with antigen conformational stability [57]. The influence of conformational fluctuations on epitope availability of envelope proteins of HIV, influenza, and flaviviruses has also been described [58–60]. Thus, HAs from influenza viruses that are more sensitive to cross-neutralizing antibodies may be useful in vaccine strategies to generate broadly neutralizing stem antibodies.

## Supporting information

S1 Fig Amino acids sequences of MAb 4C2 heavy chain and light chain. The Amino acids sequences of MAb 4C2 heavy chain and light chain with color coded FR, CDR and Constant regions are shown.
(EPS)

## References

[1] Quinones-Parra S, Loh L, Brown LE, Kedzierska K, Valkenburg SA. Universal immunity to influenza must outwit immune evasion. Front Microbiol. 2014;5:285 10.3389/fmicb.2014.00285 24971078 PMC4054793

[2] Xu R, Ekiert DC, Krause JC, Hai R, Crowe JE Jr., Wilson IA. Structural basis of preexisting immunity to the 2009 H1N1 pandemic influenza virus. Science. 2010;328(5976):357–60. 10.1126/science.1186430 20339031 PMC2897825

[3] Hensley SE, Das SR, Bailey AL, Schmidt LM, Hickman HD, Jayaraman A, et al Hemagglutinin receptor binding avidity drives influenza A virus antigenic drift. Science. 2009;326(5953):734–6. 10.1126/science.1178258 19900932 PMC2784927

[4] Yewdell JW, Caton AJ, Gerhard W. Selection of influenza A virus adsorptive mutants by growth in the presence of a mixture of monoclonal antihemagglutinin antibodies. J Virol. 1986;57(2):623–8. 2418215 10.1128/jvi.57.2.623-628.1986 PMC252777

[5] Corti D, Voss J, Gamblin SJ, Codoni G, Macagno A, Jarrossay D, et al A neutralizing antibody selected from plasma cells that binds to group 1 and group 2 influenza A hemagglutinins. Science. 2011;333(6044):850–6. 10.1126/science.1205669 21798894

[6] Dreyfus C, Laursen NS, Kwaks T, Zuijdgeest D, Khayat R, Ekiert DC, et al Highly conserved protective epitopes on influenza B viruses. Science. 2012;337(6100):1343–8. 10.1126/science.1222908 22878502 PMC3538841

[7] Ekiert DC, Bhabha G, Elsliger MA, Friesen RH, Jongeneelen M, Throsby M, et al Antibody recognition of a highly conserved influenza virus epitope. Science. 2009;324(5924):246–51. 10.1126/science.1171491 19251591 PMC2758658

[8] Ekiert DC, Friesen RH, Bhabha G, Kwaks T, Jongeneelen M, Yu W, et al A highly conserved neutralizing epitope on group 2 influenza A viruses. Science. 2011;333(6044):843–50. 10.1126/science.1204839 21737702 PMC3210727

[9] Friesen RH, Lee PS, Stoop EJ, Hoffman RM, Ekiert DC, Bhabha G, et al A common solution to group 2 influenza virus neutralization. Proc Natl Acad Sci U S A. 2014;111(1):445–50. 10.1073/pnas.1319058110 24335589 PMC3890827

[10] Kashyap AK, Steel J, Oner AF, Dillon MA, Swale RE, Wall KM, et al Combinatorial antibody libraries from survivors of the Turkish H5N1 avian influenza outbreak reveal virus neutralization strategies. Proc Natl Acad Sci U S A. 2008;105(16):5986–91. 10.1073/pnas.0801367105 18413603 PMC2329690

[11] Okuno Y, Isegawa Y, Sasao F, Ueda S. A common neutralizing epitope conserved between the hemagglutinins of influenza A virus H1 and H2 strains. J Virol. 1993;67(5):2552–8. 7682624 10.1128/jvi.67.5.2552-2558.1993 PMC237575

[12] Smirnov YA, Lipatov AS, Gitelman AK, Okuno Y, Van Beek R, Osterhaus AD, et al An epitope shared by the hemagglutinins of H1, H2, H5, and H6 subtypes of influenza A virus. Acta Virol. 1999;43(4):237–44. 10749369

[13] Sui J, Hwang WC, Perez S, Wei G, Aird D, Chen LM, et al Structural and functional bases for broad-spectrum neutralization of avian and human influenza A viruses. Nat Struct Mol Biol. 2009;16(3):265–73. 10.1038/nsmb.1566 19234466 PMC2692245

[14] Throsby M, van den Brink E, Jongeneelen M, Poon LL, Alard P, Cornelissen L, et al Heterosubtypic neutralizing monoclonal antibodies cross-protective against H5N1 and H1N1 recovered from human IgM+ memory B cells. PLoS One. 2008;3(12):e3942 10.1371/journal.pone.0003942 19079604 PMC2596486

[15] Sui J, Sheehan J, Hwang WC, Bankston LA, Burchett SK, Huang CY, et al Wide prevalence of heterosubtypic broadly neutralizing human anti-influenza A antibodies. Clinical infectious diseases: an official publication of the Infectious Diseases Society of America. 2011;52(8):1003–9. 21460314 10.1093/cid/cir121 PMC3070035

[16] Wang W, Alvarado-Facundo E, Chen Q, Anderson CM, Scott D, Vassell R, et al Serum Samples From Middle-aged Adults Vaccinated Annually with Seasonal Influenza Vaccines Cross-neutralize Some Potential Pandemic Influenza Viruses. J Infect Dis. 2016;213(3):403–6. 10.1093/infdis/jiv407 26243315 PMC7313900

[17] Wang W, Song HS, Keller PW, Alvarado-Facundo E, Vassell R, Weiss CD. Conformational Stability of the Hemagglutinin of H5N1 Influenza A Viruses Influences Susceptibility to Broadly Neutralizing Stem Antibodies. J Virol. 2018;92(12). 10.1128/JVI.00247-18 PMC5974491 29593038

[18] Neumann G, Watanabe T, Ito H, Watanabe S, Goto H, Gao P, et al Generation of influenza A viruses entirely from cloned cDNAs. Proc Natl Acad Sci U S A. 1999;96(16):9345–50. 10.1073/pnas.96.16.9345 10430945 PMC17785

[19] Hoffmann E, Krauss S, Perez D, Webby R, Webster RG. Eight-plasmid system for rapid generation of influenza virus vaccines. Vaccine. 2002;20(25–26):3165–70. 12163268 10.1016/s0264-410x(02)00268-2

[20] Hoffmann E, Neumann G, Kawaoka Y, Hobom G, Webster RG. A DNA transfection system for generation of influenza A virus from eight plasmids. Proc Natl Acad Sci U S A. 2000;97(11):6108–13. 10.1073/pnas.100133697 10801978 PMC18566

[21] Magadan JG, Khurana S, Das SR, Frank GM, Stevens J, Golding H, et al Influenza A virus hemagglutinin trimerization completes monomer folding and antigenicity. J Virol. 2013;87(17):9742–53. 10.1128/JVI.00471-13 23824811 PMC3754138

[22] Yewdell JW, Yellen A, Bachi T. Monoclonal antibodies localize events in the folding, assembly, and intracellular transport of the influenza virus hemagglutinin glycoprotein. Cell. 1988;52(6):843–52. 2450677 10.1016/0092-8674(88)90426-6

[23] Yewdell JW, Gerhard W, Bachi T. Monoclonal anti-hemagglutinin antibodies detect irreversible antigenic alterations that coincide with the acid activation of influenza virus A/PR/834-mediated hemolysis. J Virol. 1983;48(1):239–48. 6193286 10.1128/jvi.48.1.239-248.1983 PMC255340

[24] Abacioglu YH, Fouts TR, Laman JD, Claassen E, Pincus SH, Moore JP, et al Epitope mapping and topology of baculovirus-expressed HIV-1 gp160 determined with a panel of murine monoclonal antibodies. AIDS Res Hum Retroviruses. 1994;10(4):371–81. 8068416 10.1089/aid.1994.10.371

[25] Wang W, Butler EN, Veguilla V, Vassell R, Thomas JT, Moos M Jr., et al Establishment of retroviral pseudotypes with influenza hemagglutinins from H1, H3, and H5 subtypes for sensitive and specific detection of neutralizing antibodies. Journal of virological methods. 2008;153(2):111–9. 10.1016/j.jviromet.2008.07.015 18722473

[26] Wang W, Anderson CM, De Feo CJ, Zhuang M, Yang H, Vassell R, et al Cross-neutralizing antibodies to pandemic 2009 H1N1 and recent seasonal H1N1 influenza A strains influenced by a mutation in hemagglutinin subunit 2. PLoS pathogens. 2011;7(6):e1002081 10.1371/journal.ppat.1002081 21695241 PMC3111540

[27] Wang W, Xie H, Ye Z, Vassell R, Weiss CD. Characterization of lentiviral pseudotypes with influenza H5N1 hemagglutinin and their performance in neutralization assays. Journal of virological methods. 2010;165(2):305–10. 10.1016/j.jviromet.2010.02.009 20153374

[28] Bottcher E, Matrosovich T, Beyerle M, Klenk HD, Garten W, Matrosovich M. Proteolytic activation of influenza viruses by serine proteases TMPRSS2 and HAT from human airway epithelium. J Virol. 2006;80(19):9896–8. 10.1128/JVI.01118-06 16973594 PMC1617224

[29] Alvarado-Facundo E, Gao Y, Ribas-Aparicio RM, Jimenez-Alberto A, Weiss CD, Wang W. Influenza virus M2 protein ion channel activity helps to maintain pandemic 2009 H1N1 virus hemagglutinin fusion competence during transport to the cell surface. J Virol. 2015;89(4):1975–85. 10.1128/JVI.03253-14 25473053 PMC4338904

[30] Wang W, Castelan-Vega JA, Jimenez-Alberto A, Vassell R, Ye Z, Weiss CD. A mutation in the receptor binding site enhances infectivity of 2009 H1N1 influenza hemagglutinin pseudotypes without changing antigenicity. Virology. 2010;407(2):374–80. 10.1016/j.virol.2010.08.027 20869738

[31] Wang W, Chen Q, Ford-Siltz LA, Katzelnick LC, Parra GI, Song HS, et al Neutralizing Antibody Responses to Homologous and Heterologous H1 and H3 Influenza A Strains after Vaccination with Inactivated Trivalent Influenza Vaccine Vary with Age and Prior Year Vaccination. Clinical infectious diseases: an official publication of the Infectious Diseases Society of America. 2018. 10.1093/cid/ciy818 30256912

[32] Meseda CA, Atukorale V, Soto J, Eichelberger MC, Gao J, Wang W, et al Immunogenicity and Protection Against Influenza H7N3 in Mice by Modified Vaccinia Virus Ankara Vectors Expressing Influenza Virus Hemagglutinin or Neuraminidase. Scientific reports. 2018;8(1):5364 10.1038/s41598-018-23712-9 29599502 PMC5876369

[33] Boulay F, Doms RW, Wilson I, Helenius A. The influenza hemagglutinin precursor as an acid-sensitive probe of the biosynthetic pathway. EMBO J. 1987;6(9):2643–50. 3315651 10.1002/j.1460-2075.1987.tb02555.x PMC553685

[34] Ruigrok RW, Cremers AF, Beyer WE, de Ronde-Verloop FM. Changes in the morphology of influenza particles induced at low pH. Arch Virol. 1984;82(3–4):181–94. 6508530 10.1007/BF01311162

[35] Skehel JJ, Bayley PM, Brown EB, Martin SR, Waterfield MD, White JM, et al Changes in the conformation of influenza virus hemagglutinin at the pH optimum of virus-mediated membrane fusion. Proc Natl Acad Sci U S A. 1982;79(4):968–72. 10.1073/pnas.79.4.968 6951181 PMC345880

[36] Vanderlinden E, Goktas F, Cesur Z, Froeyen M, Reed ML, Russell CJ, et al Novel inhibitors of influenza virus fusion: structure-activity relationship and interaction with the viral hemagglutinin. J Virol. 2010;84(9):4277–88. 10.1128/JVI.02325-09 20181685 PMC2863778

[37] Xu R, Wilson IA. Structural characterization of an early fusion intermediate of influenza virus hemagglutinin. J Virol. 2011;85(10):5172–82. 10.1128/JVI.02430-10 21367895 PMC3126156

[38] Wang W, DeFeo CJ, Alvarado-Facundo E, Vassell R, Weiss CD. Intermonomer Interactions in Hemagglutinin Subunits HA1 and HA2 Affecting Hemagglutinin Stability and Influenza Virus Infectivity. J Virol. 2015;89(20):10602–11. 10.1128/JVI.00939-15 26269180 PMC4580181

[39] Alvarado-Facundo E, Vassell R, Schmeisser F, Weir JP, Weiss CD, Wang W. Glycosylation of Residue 141 of Subtype H7 Influenza A Hemagglutinin (HA) Affects HA-Pseudovirus Infectivity and Sensitivity to Site A Neutralizing Antibodies. PLoS One. 2016;11(2):e0149149 10.1371/journal.pone.0149149 26862918 PMC4749315

[40] Matsuda K, Huang J, Zhou T, Sheng Z, Kang BH, Ishida E, et al Prolonged evolution of the memory B cell response induced by a replicating adenovirus-influenza H5 vaccine. Sci Immunol. 2019;4(34). 10.1126/sciimmunol.aau2710 31004012

[41] DiLillo DJ, Tan GS, Palese P, Ravetch JV. Broadly neutralizing hemagglutinin stalk-specific antibodies require FcgammaR interactions for protection against influenza virus in vivo. Nature medicine. 2014;20(2):143–51. 10.1038/nm.3443 24412922 PMC3966466

[42] Cox F, Kwaks T, Brandenburg B, Koldijk MH, Klaren V, Smal B, et al HA Antibody-Mediated FcgammaRIIIa Activity Is Both Dependent on FcR Engagement and Interactions between HA and Sialic Acids. Frontiers in immunology. 2016;7:399 10.3389/fimmu.2016.00399 27746785 PMC5040702

[43] Palese P, Wang TT. Why do influenza virus subtypes die out? A hypothesis. MBio. 2011;2(5). 10.1128/mBio.00150-11 PMC3163940 21878571

[44] Ellebedy AH, Krammer F, Li GM, Miller MS, Chiu C, Wrammert J, et al Induction of broadly cross-reactive antibody responses to the influenza HA stem region following H5N1 vaccination in humans. Proc Natl Acad Sci U S A. 2014;111(36):13133–8. 10.1073/pnas.1414070111 25157133 PMC4246941

[45] Nachbagauer R, Wohlbold TJ, Hirsh A, Hai R, Sjursen H, Palese P, et al Induction of broadly reactive anti-hemagglutinin stalk antibodies by an H5N1 vaccine in humans. J Virol. 2014;88(22):13260–8. 10.1128/JVI.02133-14 25210189 PMC4249097

[46] Whittle JR, Wheatley AK, Wu L, Lingwood D, Kanekiyo M, Ma SS, et al Flow cytometry reveals that H5N1 vaccination elicits cross-reactive stem-directed antibodies from multiple Ig heavy-chain lineages. J Virol. 2014;88(8):4047–57. 10.1128/JVI.03422-13 24501410 PMC3993745

[47] Chai N, Swem LR, Reichelt M, Chen-Harris H, Luis E, Park S, et al Two Escape Mechanisms of Influenza A Virus to a Broadly Neutralizing Stalk-Binding Antibody. PLoS pathogens. 2016;12(6):e1005702 10.1371/journal.ppat.1005702 27351973 PMC4924800

[48] Yamayoshi S, Yasuhara A, Ito M, Uraki R, Kawaoka Y. Differences in the ease with which mutant viruses escape from human monoclonal antibodies against the HA stem of influenza A virus. Journal of clinical virology: the official publication of the Pan American Society for Clinical Virology. 2018;108:105–11. 30292135 10.1016/j.jcv.2018.09.016 PMC12973287

[49] Doud MB, Lee JM, Bloom JD. How single mutations affect viral escape from broad and narrow antibodies to H1 influenza hemagglutinin. Nature communications. 2018;9(1):1386 10.1038/s41467-018-03665-3 29643370 PMC5895760

[50] Dreyfus C, Ekiert DC, Wilson IA. Structure of a classical broadly neutralizing stem antibody in complex with a pandemic H2 influenza virus hemagglutinin. J Virol. 2013;87(12):7149–54. 10.1128/JVI.02975-12 23552413 PMC3676097

[51] Okuno Y, Matsumoto K, Isegawa Y, Ueda S. Protection against the mouse-adapted A/FM/1/47 strain of influenza A virus in mice by a monoclonal antibody with cross-neutralizing activity among H1 and H2 strains. J Virol. 1994;68(1):517–20. 8254764 10.1128/jvi.68.1.517-520.1994 PMC236314

[52] Lipatov AS, Gitelman AK, Smirnov Yu A. Prevention and treatment of lethal influenza A virus bronchopneumonia in mice by monoclonal antibody against haemagglutinin stem region. Acta Virol. 1997;41(6):337–40. 9607093

[53] Schneemann A, Speir JA, Tan GS, Khayat R, Ekiert DC, Matsuoka Y, et al A virus-like particle that elicits cross-reactive antibodies to the conserved stem of influenza virus hemagglutinin. J Virol. 2012;86(21):11686–97. 10.1128/JVI.01694-12 22896619 PMC3486276

[54] Smirnov YA, Lipatov AS, Gitelman AK, Claas EC, Osterhaus AD. Prevention and treatment of bronchopneumonia in mice caused by mouse-adapted variant of avian H5N2 influenza A virus using monoclonal antibody against conserved epitope in the HA stem region. Arch Virol. 2000;145(8):1733–41. 11003481 10.1007/s007050070088

[55] Henry Dunand CJ, Leon PE, Kaur K, Tan GS, Zheng NY, Andrews S, et al Preexisting human antibodies neutralize recently emerged H7N9 influenza strains. J Clin Invest. 2015;125(3):1255–68. 10.1172/JCI74374 25689254 PMC4362269

[56] Prachanronarong KL, Canale AS, Liu P, Somasundaran M, Hou S, Poh YP, et al Mutations in Influenza A Virus Neuraminidase and Hemagglutinin Confer Resistance against a Broadly Neutralizing Hemagglutinin Stem Antibody. J Virol. 2019;93(2). 10.1128/JVI.01639-18 PMC6321927 30381484

[57] Thai R, Moine G, Desmadril M, Servent D, Tarride JL, Menez A, et al Antigen stability controls antigen presentation. The Journal of biological chemistry. 2004;279(48):50257–66. 15364925 10.1074/jbc.M405738200

[58] Kwong PD, Doyle ML, Casper DJ, Cicala C, Leavitt SA, Majeed S, et al HIV-1 evades antibody-mediated neutralization through conformational masking of receptor-binding sites. Nature. 2002;420(6916):678–82. 12478295 10.1038/nature01188

[59] Kuhn RJ, Dowd KA, Beth Post C, Pierson TC. Shake, rattle, and roll: Impact of the dynamics of flavivirus particles on their interactions with the host. Virology. 2015;479–480:508–17. 10.1016/j.virol.2015.03.025 25835729 PMC4900690

[60] Bachi T, Gerhard W, Yewdell JW. Monoclonal antibodies detect different forms of influenza virus hemagglutinin during viral penetration and biosynthesis. J Virol. 1985;55(2):307–13. 2410628 10.1128/jvi.55.2.307-313.1985 PMC254934

